# Recommendations for Implementing Policy, Systems, and Environmental Improvements to Address Chronic Diseases in Asian Americans, Native Hawaiians, and Pacific Islanders

**DOI:** 10.5888/pcd11.140272

**Published:** 2014-11-20

**Authors:** Pedro Arista, Ed Tepporn, Simona Kwon, Catlin Rideout, Shilpa Patel, Marianne Chung, Roxanna Bautista, Chau Trinh-Shevrin, Kathy Ko-Chin

**Affiliations:** Author Affiliations: Ed Tepporn, Marianne Chung, Roxanna Bautista, Kathy Ko-Chin, Asian & Pacific Islander American Health Forum, San Francisco, California; Simona Kwon, Catlin Rideout, Shilpa Patel, Chau Trinh-Shevrin, New York University School of Medicine, Department of Population Health, New York, New York.

## Abstract

Emphasis has increased recently on disseminating high-impact, population-wide strategies for the prevention of chronic diseases. However, such strategies are typically not effective at reaching Asian Americans, Native Hawaiians, Pacific Islanders, or other underserved communities. The objectives of this article were to 1) present the methods of the Strategies to Reach and Implement the Vision of Health Equity program in which 15 community-based organizations in the United States and the Pacific region implemented evidence-based policy, systems, and environmental improvements in their local communities and 2) provide recommendations for using these tailored approaches in other communities and geographic locations. Further support is needed for organizations in tailoring these types of population-wide strategies. Implementing population health improvements should be adapted to maximize effectiveness to decrease chronic diseases in these populations and ultimately eliminate racial/ethnic health disparities.

## Introduction

Asian Americans and Native Hawaiians and Pacific Islanders (NHPIs) are a rapidly growing population in the United States (1). They encompass a diverse spectrum of more than 50 countries (eg, China, Philippines, Samoa) and speak more than 100 languages and dialects (1). Although Asian Americans and NHPIs are regarded as a “model minority community” with universally high levels of education and wealth, recent census data indicate otherwise (2).

Gaps in the literature and a lack of disaggregated data on nutrition and physical activity prevent in-depth understanding of the health of Asian Americans and NHPIs. However, some research does indicate poor nutrition patterns for certain Asian American and NHPI subgroups, including Chinese, Filipinos, Japanese, and Native Hawaiians (3). Similarly, studies suggest that contributors to poor nutrition among Asian Americans and NHPIs are the convenience and availability of American fast food (4), lack of access to traditional foods, and exposure to Western-style diets and unhealthy options (5).

Lack of physical activity also appears to be a challenge in these populations. For example, nearly 48% of Asian Americans do not meet US guidelines for aerobic or muscle-strengthening activity (6). The Youth Risk Behavior Surveillance System indicates that nearly 60% of Asian American and 54% of NHPI youth were not physically active at least 60 minutes per day on 5 or more days (7). Several studies identified busy work schedules, lack of economic resources or transportation to exercise facilities, and neighborhood safety as concerns preventing members of underserved racial/ethnic communities from engaging in physical activity (8).

The Centers for Disease Control and Prevention (CDC) encourages the dissemination of evidence-based strategies that use a policy, systems, and environmental (PSE) improvement approach (9) for the prevention of chronic diseases (10,11). PSE improvements move away from focusing on individual behavior to improve health and instead focus on changing policies, systems, and the physical environment (12). PSE improvements aim to make large, population-wide impacts (12). However, such strategies are typically not effective at reaching racial/ethnic minority groups such as Asian Americans and NHPIs, who may be socially and linguistically isolated from mainstream campaigns and programs (13,14). To help address this gap, from February 2013 through September 2013, the Asian & Pacific Islander American Health Forum (APIAHF), in partnership with the New York University Center for the Study of Asian American Health (CSAAH) developed the Strategies to Reach and Implement the Vision of Health Equity (STRIVE) program to reduce chronic disease risk factors in Asian American and NHPI communities. Funded through CDC’s Racial and Ethnic Approaches to Community Health (REACH) cooperative agreement award, the STRIVE program was 1 of 6 awardees charged with distributing at least 75% of funds to collectively support 90 organizations to implement PSE improvements.

The objective of this article was to present the methods of the STRIVE program in which community-based organizations (CBOs) implemented PSE improvements in their local communities and provide recommendations for using these tailored approaches in other communities.

## The STRIVE Program and Grant Application Process

The aim of the STRIVE program was to reduce chronic disease health disparities among Asian Americans and NHPIs by implementing PSE improvements focused on physical activity and healthy eating. The 3 primary goals for the program were

1. To ensure meaningful involvement and input from Asian American and NHPI communities in the development and implementation of a community action plan to address chronic disease health disparities;
2. To increase the ability of CBOs and multisector partnerships to implement evidence-based PSE improvements that reduce health disparities among Asian American and NHPI communities that have a high burden of risk factors related to physical activity, poor nutrition, and weight management; and
3. To strengthen and expand a national network of Asian American and NHPI stakeholders focused on PSE improvements through training and technical assistance.

To achieve these goals and manage the program, APIAHF and CSAAH forged a co-lead structure in which each institution leveraged its expertise and strengths to provide training and technical assistance to the program and program staff served as project officers. Project officers were the primary liaison to the CBOs, tracking and monitoring CBOs’ deliverables and performance (eg, monthly calls, progress reports, site visits) and providing training and technical assistance.

STRIVE launched a competitive request-for-proposal process (November 1, 2012, to January 2, 2013). Reviewers independently reviewed 17 proposals and rated the following 3 criteria: understanding of community needs related to physical activity and nutrition, experience in partnering with multiple sectors, and experience in addressing health disparities through population-based strategies. Given the short time frame of the program, the review committee also evaluated organizational capacity to implement PSE improvements. In addition, CDC’s geographic funding preferences and coordination with the other 5 REACH national awardees were considered to maximize impact across the country and minimize geographic overlap. The committee selected 15 CBOs and, on February 22, 2013, distributed $3 million in grants ranging from $75,000 to $300,000; program funding ended September 29, 2013. Funded organizations had 7 months to implement their proposed projects. 

**Figure:**
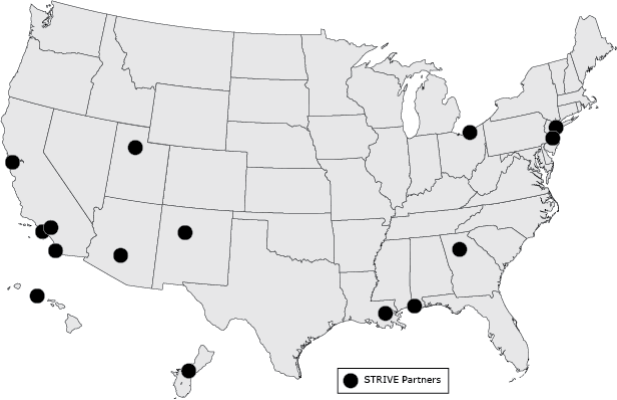
Location of partners in Strategies to Reach and Implement the Vision of Health Equity (STRIVE) program, February–September 2013. The 15 partners reached Asian American, Native Hawaiian, and Pacific Islander populations in 11 states and Guam; strategies focused on improving nutrition, physical activity, or a combination thereof.

As part of its grant application, each CBO identified a jurisdiction (eg, city, school district, county) and a target population. The STRIVE program identified 2 levels of reach for each CBO. The first level was a PSE improvement that would reach at least 75% of the target population in the jurisdiction. This percentage was calculated by dividing the number of Asian Americans and NHPIs reached by a PSE improvement by the total number of Asian Americans and NHPIs in the jurisdiction. For example, estimates of the potential reach of nutrition policies in faith-based institutions were calculated by dividing the number of Asian Americans and NHPIs who could be reached through the institutions by the total number of Asian Americans and NHPIs in the jurisdiction. Reach estimates were verified by program staff using publicly available data sources, such as the US Census 2010. For the second level of reach, because the strategies implemented by the CBOs were population-based strategies, CDC anticipated that PSE improvements would also have an impact beyond the CBOs’ jurisdictions and target populations.

The grant application also required each CBO to include a plan for the following: enhancing a multisector partnership; conducting a community health assessment and policy scan; developing and implementing a community action plan with PSE improvements.

**Enhancing multisector partnerships. **Each CBO enhanced a multisector partnership within the first month of its project. This partnership helped achieve the first program goal to support engagement of community stakeholders across multiple sectors. The partnership was central to planning and implementing the overall project for each organization and could include representatives from such areas as public health, education, economic development, transportation, faith-based communities, or the business sector. Multisector partnerships have been shown to improve population health (15,16).

**Conducting a community health assessment and policy scan.** To achieve the second goal, to prepare CBOs to implement strategies that reduce health disparities among Asian American and NHPI populations, each organization completed a community health assessment and policy scan within the first 2 months of funding by using the Community Health Assessment aNd Group Evaluation (CHANGE) tool. The tool was designed by CDC to help communities gather and organize data on assets and areas for improvement before deciding on critical issues identified in a community action plan (17). Through the collection of data from various sources, the tool provides a community snapshot of potential PSE improvements and helps to identify areas of buy-in among the multisector partnership.

**Developing and implementing a community action plan with PSE improvements.** Within the first 4 months of funding, each CBO worked closely with partners to develop a community action plan that included PSE improvements informed by data collected by the CHANGE tool. CDC technical experts provided input to ensure that the PSE improvements selected by each CBO would have the desired impact on health, reach the jurisdiction, address special considerations for Asian American and NHPI populations, and comply with anti-lobbying provisions.

## Program Outcomes

During the 7-month program period from February through September 2013, the 15 CBOs potentially reached 582,590 Asian Americans and NHPIs (Table). CBOs selected strategies that focused on nutrition or physical activity or a combination thereof. Eight CBOs focused on nutrition and had the potential to reach 303,942 Asian Americans and NHPIs (Table). Improvement strategies varied; several strategies included the development of multiple community gardens, one increased access to the Supplemental Nutrition Assistance Program at farmers markets, and others implemented institutional nutrition-related policies, such as offering at least one healthy option at communal meals. The PSE improvements took place in various settings: apartment houses, Asian American and NHPI restaurants, and faith-based institutions. Several CBOs targeted subgroups, such as Filipinos, Laotians, Native Hawaiians, Samoans, and Vietnamese.

**Table:** Policy, Systems, and Environmental (PSE) Improvements Implemented by Partners in the Strategies to Reach and Implement the Vision of Health Equity (STRIVE) Program, 2013

Partner	Target Population	Location	Potential Population Reach^a^ (N = 582,590)	Total Population in Jurisdiction^b^ (N = 25,284,931)	PSE Improvement
Asian Pacific Community in Action	Asian American	Chandler, Arizona	18,500	388,893	Community gardens: the gardens included vegetables and greens (eg, bok choy, Chinese okra, daikon, long bean, mizuna) that are in many Asian American recipes.
Boat People–Alabama	Asian American, Native Hawaiian, and Pacific Islander	Bayou La Batre, Alabama	2,646	2,646	Farmers market: the vendors from the market provided fresh fish and shellfish at a reduced price as well as greens and herbs, including red mustard, mizspoona, snap peas, Thai basil, and kale.
Center for Pan Asian Community Services, Inc.	Asian American	DeKalb and Gwinnett counties, Georgia	96,312	694,671 in DeKalb County; 810,624 in Gwinnett County	Community gardens: the gardens grew vegetables and herbs (bok choy, bitter melon, edamame, long bean) that are in many Asian American recipes.
Korean Community Services	Korean and Korean American church attendees	Palisades Park, New Jersey	8,910	19,561	Policy at faith-based institutions: the policy increased access to healthier food options, including brown/multigrain rice and multigrain/whole wheat bread.
Kōkua Kalihi Valley Comprehensive Family Services	Filipino, Native Hawaiian, and Pacific Islander	Honolulu, specifically Kalihi Valley, Hawaii	18,893	955,215	Community gardens and farmers markets: the gardens and markets included fruits and vegetables (eg, taro, mango, certain greens) that are relevant to the target populations. Also increased access to Supplemental Nutrition Assistance Program at targeted markets. Education materials were translated into Chuukese, Samoan, and Ilokano languages.
Operation Samahan	Filipino, Native Hawaiian, Laotian, Vietnamese, and Samoan restaurant patrons	San Diego, California	141,528	3,100,500	Restaurant policy: implemented a policy in Filipino, Vietnamese, Thai, Laotian, Chamorro and Hawaiian restaurants to change their menus to include healthier ingredients (fruits and vegetables), small portion options, and nutrition labeling.
Restaurant Opportunities Center of New Orleans	Vietnamese restaurant workers	New Orleans, Louisiana	9,750	341,407	Restaurant policy: implemented a policy in Vietnamese restaurants to increase healthy food options for restaurant workers and their families.
Taulama for Tongans	Pacific Islander church attendees	San Mateo, California	7,403	721,183	Policy at faith-based institutions: implemented a policy to change their menus for community events to include healthier foods that are relevant to Pacific Islanders as well as provide smaller portions.
Boat People–California	Vietnamese nail salon workers	Orange County, California	13,342	3,021,840	Worksite policy: nail salon businesses adopted a culturally appropriate policy for physical activity exercises. Provided cultural competency training to staff and translated materials.
National Tongan American Society	Native Hawaiian and Pacific Islander	Salt Lake County, Utah	9,751	1,032,226	Joint-use agreements: schools and faith-based institutions reaching Native Hawaiians and Pacific Islanders implemented joint-use agreements increasing access to free environments for physical activity. Physical activities included hula and Zumba.
New Mexico Asian Family Center	Asian American	Albuquerque, New Mexico	14,347	545,083	Walking trails: stakeholders including the health department, faith-based institutions, and small businesses adopted and created a walking trail in the International District, a community heavily populated by Asian Americans. Provided translated materials in Chinese and Vietnamese.
Asian Services In Action, Inc.	Asian American	Cleveland/Cuyahoga County, Ohio	97,600	397,972 in Cleveland; 1,278,024 in Cuyahoga County	Physical activity and nutrition policies: the master planning committee of Asia Town adopted changes in the environmental plan to designate green space for physical activity, including an increase in walking trails and bicycle lanes for community residents. Refugee resettlement organizations and cultural community schools incorporated nutrition information into their acculturation courses to emphasize the benefits of healthy foods and detriment of fast foods. Ethnic voluntary social groups adopted policies to have nutritional standards during community events.
Orange County Asian and Pacific Islander Community Alliance	Asian American, Native Hawaiian and Pacific Islander youth	Orange County, California	74,297	3,021,840	School policy: school districts adopted a policy to disseminate information on free and reduced-price lunch. Developed an informational sheet about the target populations and Healthy Eating Active Living to be inserted into the Get Fit tool kits disseminated by the schools. Translation services were provided to families, and materials were translated into various languages, including Chinese, Filipino, and Korean.
United Sikhs	Sikh gurdwara attendees	New Jersey	33,255	8,793,888	Policies at faith-based institutions: gurdwaras implemented policies for healthy food options during langar, including smaller portions and lower levels of trans fat. Gurdwaras also encouraged physical activity before the beginning of prayer. A gurdwara is the place of worship for Sikhs, and langar is a tradition of serving food in a gurdwara to all visitors (regardless of racial/ethnic or religious background) for free. Sikh Khalsa schools (cultural community schools) also developed and implemented a nutrition curriculum for students.
University of Hawai**’**i Manoa^c^	Asian American, Native Hawaiian and Pacific Islander residents and Guam government employees	Guam	36,056	159,358	Worksite wellness policy: increased the number of Guam Government Departments that participated in a Worksite Wellness program aimed to reach full-time employees. Community Gardens in villages: increased the number of community gardens that included popular foods for Asian and Pacific Islander dishes.

a Number of Asian Americans, Native Hawaiians, and Pacific Islanders in the jurisdiction who could be reached by the partner. Reach estimates were verified by community-based organization staff by using publicly available data sources, such as the US Census 2010 (2).

b Sources: US Census Bureau (2), 2012 American Community Survey 5-year Estimates (18), and 2010 Guam Summary File (19).

c The University of Hawai’i Manoa has a history of working with the US Associated Pacific Islands, which includes Guam. Through the University, Guam’s Non-Communicable Disease Consortium implemented its STRIVE Project.

Three CBOs selected PSE improvements that focused on physical activity and had the potential to reach 37,440 Asian Americans and NHPIs. These strategies included development of worksite policies to create spaces for physical activity, development and promotion of walking trails, and the implementation of joint-use agreements to increase access to physical activity. PSE improvements took place in such sites as nail salons, faith-based institutions, schools, and neighborhoods frequented by Asian Americans and NHPIs (eg, the International District in Albuquerque, New Mexico). Several CBOs targeted subgroups such as Fijians, Samoans, Tongans, and Vietnamese.

Four CBOs elected to implement PSE improvements related to both nutrition and physical activity. These CBOs had the potential to reach 241,208 Asian Americans and NHPIs. PSE improvements included working with a city master planning group to guide the development of bicycle lanes in a community with a large Asian American population (Asia Town in Cleveland, Ohio), developing worksite policies to create spaces for physical activity, and working with cultural community schools (eg, Sikh Khalsa schools) to develop and implement a nutrition curriculum for students. PSE improvements took place at various sites: schools, faith-based institutions, cultural or language schools, and refugee community centers. Several CBOs focused on population subgroups, including Chamorro, Korean, Sikh, and Vietnamese.

## Recommendations

The following 5 key recommendations build on lessons learned during the STRIVE program and should be considered by organizations working with Asian Americans and NHPIs to implement PSE improvements or similar population-wide health strategies.

### 1. Involve CBOs to achieve PSE improvements

CBOs play critical roles in their local communities and across multiple sectors. Their history of implementing interventions makes them well-positioned to navigate local culture and adapt strategies to the needs of their communities. Also, these organizations have strong relationships with local community members. Because they often serve as gatekeepers, their involvement can be vital to successful implementation of PSE improvements. Hard-to-reach communities may also be more likely to go to CBOs to access culturally and linguistically appropriate information and services because trust has been established (20).

### 2. Select CBOs with the capacity and infrastructure to implement population-wide strategies

It is essential to fund organizations that have the capacity and systems in place to implement population-wide strategies. Organizational capacity to implement PSE improvements was a criterion for selection during the request-for-proposal process, and it was especially critical because of the short time frame for CBOs to implement their projects.

### 3. Provide culturally sensitive training and technical assistance

PSE improvement is a relatively new concept for CBOs serving Asian Americans and NHPIs. Project officers found it was important to provide culturally sensitive training and technical assistance to increase CBOs’ understanding of and skills in the PSE planning and implementation processes. For example, CBOs were given specialized training to implement the CHANGE assessment tool in their communities. The findings from the tool guided the development and implementation of a community action plan.

### 4. Collect and analyze disaggregated data

The collection and analysis of disaggregated data for Asian Americans and NHPIs is essential to understand the health disparities and needs of these populations, which are diverse in ethnicity, culture, and language. Because many publicly funded entities (eg, health departments, hospitals) do not collect disaggregated data, the CBOs in our program often used other sources of disaggregated data, including quantitative data from nontraditional sources such as churches (eg, membership data) and businesses (eg, number of nail salon workers). CBOs also conducted key informant interviews with community stakeholders. 

### 5. Use a co-lead structure as a means to implement project

APIAHF and CSAAH forged a co-lead structure that involved staff in the leadership and management of the program. Moreover, these 2 institutions harnessed their own expertise and strengths. APIAHF’s experience in funding CBOs to implement public health interventions in various disease areas and CSAAH’s expertise in community-based participatory research was critical in developing and delivering technical assistance to develop Asian American and NHPI multisector partnerships across the country into a national network. Additionally, this partnership had systems and an infrastructure in place to support CBOs. Lastly, this combined partnership allowed each institution to have access to a larger network of CBOs serving Asian Americans and NHPIs.

## Other Considerations

This analysis of the STRIVE program has at least 2 limitations. First, disaggregated data for Asian Americans and NHPIs were lacking at the community level. Second, much of the data for the community health and policy scan and for measuring reach were not up-to-date. Because of these limitations, the data on reach in this analysis are only estimates.

Nonetheless, as part of the STRIVE program, organizations implemented PSE strategies to reach a substantial proportion of Asian Americans and NHPIs in their communities. As public health resources become more constrained and health disparities in certain disease areas (eg, HIV, diabetes) increase among Asian Americans and NHPIs (21,22), an innovative design to support CBOs, such as the one implemented by the STRIVE program, will become increasingly important.

To accomplish the STRIVE program’s goals, the co-lead institutions selected CBOs according to their organizational capacity and estimated reach. They were first required to have a multisector partnership and use a community health needs assessment and policy scan to support the development and implementation of their PSE-focused community action plan. These preparatory steps were essential to accomplishing the third goal, to strengthen and expand a national network of Asian American and NHPI stakeholders focused on PSE improvements. Recommendations from the program demonstrate how such an approach should be adapted to maximize effectiveness and mitigate racial and ethnic health disparities.
